# Mortality Trends and Disparities in Cerebrovascular Disease Among Diabetic Population in the United States From 1999 to 2020: A CDC WONDER Analysis

**DOI:** 10.1002/edm2.70091

**Published:** 2025-08-22

**Authors:** Allahdad Khan, Waseef Ullah, Moeen Ikram, Rameez Qasim, Umama Alam, Maheen Sheraz, Ayesha Khan, Kainat Kanwal, Peter Collins, Raheel Ahmed

**Affiliations:** ^1^ Department of Medicine Nishtar Medical University Multan Pakistan; ^2^ Department of Medicine Lady Reading Hospital Peshawar Pakistan; ^3^ Frontier Medical and Dental College Abbottabad Pakistan; ^4^ Allama Iqbal Medical College Lahore Pakistan; ^5^ Khyber Medical College Peshawar Pakistan; ^6^ Continental Medical College Lahore Pakistan; ^7^ Charleston Area Medical Center, Vandalia Health Charleston West Virginia USA; ^8^ National Heart and Lung Institute Imperial College London London UK

**Keywords:** CDC WONDER, cerebrovascular disease, diabetes, mortality

## Abstract

**Background:**

Diabetes mellitus (DM) significantly increases the risk of cerebrovascular disease (CeVD), a major cause of mortality and long‐term disability. Despite improvements in healthcare, disparities in CeVD‐related mortality among diabetic populations in the United States persist.

**Methods:**

We conducted a retrospective analysis using the CDC WONDER database from 1999 to 2020 to assess mortality trends related to CeVD among adults aged ≥ 45 years with DM. Deaths were identified using ICD‐10 codes I60–I69 (CeVD) and E10–E14 (DM). Age‐adjusted mortality rates (AAMRs) were calculated, and trends were analysed using Joinpoint regression, stratified by age, race/ethnicity, geography, urbanisation, and place of death.

**Results:**

A total of 689,846 CeVD‐related deaths occurred in diabetic individuals. AAMR decreased from 36.9 in 1999 to 29.3 in 2020, with an average annual percentage change (AAPC) of −1.41%. However, a sharp rise was observed from 2018 to 2020 (APC 14.87%), indicating a concerning reversal in progress. The highest crude mortality rates were in the 75–84 age group, and the lowest in the 45–54 group. Black and Hispanic populations, rural residents, and those in the Southern United States had the highest mortality rates. The Northeast and Asian populations had the lowest, reflecting persistent disparities in access to care and preventive services.

**Conclusion:**

While CeVD mortality in diabetics declined over two decades, the recent reversal highlights emerging challenges, possibly due to healthcare disruptions and socioeconomic disparities. These findings underscore the need for targeted public health interventions to address inequities and improve outcomes in high‐risk populations.

## Graphical abstract

While cerebrovascular disease mortality in diabetics declined over two decades, the recent reversal highlights emerging challenges, possibly due to healthcare disruptions and socioeconomic disparities. These findings underscore the need for targeted public health interventions to address inequities and improve outcomes in high‐risk populations.

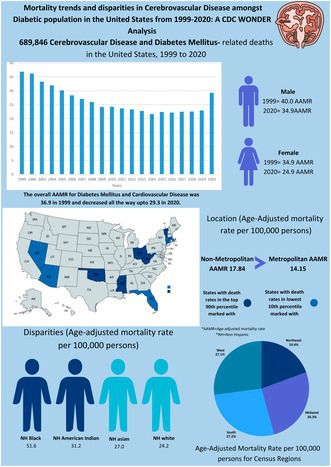

## 1 Introduction

Diabetes mellitus (DM) is a chronic metabolic disorder characterised by elevated blood glucose levels, primarily resulting from insulin resistance or impaired insulin secretion due to pancreatic dysfunction. Over time, DM can lead to a range of complex chronic complications, including macrovascular and microvascular diseases, neuropathic disorders, and diabetic foot conditions. Among these, cerebrovascular diseases (CeVD) represent some of the most severe complications in patients with DM, encompassing both ischemic and haemorrhagic strokes. The primary mechanisms underlying CeVD in patients with type 2 DM include atherosclerosis, chronic hyperglycaemia, and metabolic syndrome, all of which contribute to endothelial dysfunction and ultimately increase the risk of stroke [1].

Over 37 million adults in the United States, approximately 15% of the adult population—are currently living with DM, with an estimated additional 8 million individuals remaining undiagnosed [2]. Furthermore, the incidence of newly diagnosed DM continues to rise, with data from the Framingham Heart Study indicating a near doubling in the incidence rates among both men and women over recent decades [3]. CeVD is the fourth leading cause of death among all adults in the United States and a leading cause of death among patients with DM [4]. Among diabetic patients who survive a CeVD event (i.e., stroke) around 50% develop long‐term disability [5]. Although brain‐related complications increase with age, younger patients aged 30 to 44 years face a significantly higher risk of stroke compared to non‐diabetic individuals in the same age group [6]. The combined impact of early onset and elevated stroke risk in DM is evident in the 9% stroke prevalence among the 25.6 million adults with DM in the United States [7]. Studies have shown that patients with type 2 DM have a 1.5 to 2 fold higher risk of stroke in men and a 2 to 6.5 fold higher risk in women [8].

While several studies utilising the CDC WONDER (Centers for Disease Control and Prevention Wide‐Ranging Online Data for Epidemiologic Research) database have examined mortality trends related to stroke in the general population, limited research has specifically focused on trends in CeVD mortality among individuals with DM. Given the strong association between diabetes and an increased risk of both ischemic and haemorrhagic stroke, it is essential to assess how mortality patterns have changed over time within this high‐risk group. Such analysis can provide critical insights into the burden of CeVD among diabetic populations and inform more effective, targeted strategies for prevention and treatment.

Therefore, we evaluated the geographical and demographic variations in CeVD‐related mortality among adults aged 45 years and older with diabetes in the United States, using data from 1999 to 2020.

## 2 Methods

### 2.1 Study Setting

In this database study, death certificate data were retrieved from the CDC WONDER‐Centers for Disease Control and Prevention Wide‐Ranging OnLine Data for Epidemiologic Research database and examined from 1999 to 2020 for CeVD among the diabetic population. The International Statistical Classification of Diseases and Related Health Problems‐10th Revision (ICD‐10) as follows: I60–I69 and E10–E14.

In this descriptive study, we used the data provided by the National Center for Health Statistics (NCHS), which was made available through the Centers for Disease Control and Prevention Wide‐Ranging Online Data for Epidemiologic Research (CDC‐WONDER) Database to analyse annual mortality trends from 1999 to 2020. Data from death certificates of United States residents is annually updated on CDC‐WONDER. Using the final Multiple Cause of Death Public Use record and International Classification of Disease, 10th Revision (ICD‐10) codes: I60–I69 for CeVDs [9] and E10–E14 for DM [10], we identified death certificates listing these conditions as underlying or contributing causes of death. An institutional review board was not required for this study as it used a deidentified government‐issued public use data set. Moreover, this research was done strictly adhering to the Strengthening the Reporting of Observational Studies in Epidemiology (STROBE) guidelines [11].

### 2.2 Data Extraction

The dataset was thoroughly analysed for a wide range of demographic variables, that is, sex, range/ethnicity, age groups, regions, states, place of death, and urban–rural classification. Race/ethnicity groups were delineated as non‐Hispanics (NH) white, NH Black or African American, NH Asian or Pacific Islander, NH American Indian or Alaska Native, and Hispanics or Latinos. For age stratification, age was divided into the following categories: 45–54, 55–64, 65–74, 75–84, and 85+ years. The National Center for Health Statistics Urban–Rural Classification Scheme was used to analyse the population by urban (large metropolitan area [population > 1 million], medium/small metropolitan area [population 50,000–999,999]) and rural (population < 50,000) counties per the 2013 United States census classification. Regions were classified according to the U.S. Census Bureau definitions into Northeast, Midwest, South, and West [12]. Place of death included medical facilities (outpatient, emergency room, inpatient, death on arrival, or states unknown), home, hospice, and nursing home/long‐term care facility.

### 2.3 Statistical Analysis

Crude mortality rates (CMRs) and age‐adjusted mortality rates (AAMRs) per 100,000 related to CeVD in diabetic patients were obtained. To ensure data comparability, AAMR accounts for the population's variability in age distribution and was calculated using the direct adjustment strategy based on the 2000 standard population. In order to quantify the national annual trends, the Joinpoint Regression Program (Joinpoint V 4.9.0.0, National Cancer Institute) was employed to analyse AAMR from 1999 to 2020 [13, 14]. This technique fits log‐linear regression models when temporal variation occurs in order to identify significant changes in AAMR across time. Using two‐tailed *t*‐testing, APCs were deemed rising or falling if the slope characterising the change in mortality differed substantially from zero. *p* < 0.05 was deemed to be statistically significant.

## 3 Results

CeVD related mortality in patients with DM has resulted in a total of 689,846 deaths from 1999 to 2020, with most of the deaths occurring in the Inpatient‐Medical Facilities numbering 251,286 (36.40% of total deaths) and 35,336 deaths (5.10%) in ER or Outpatients, and 132,433 deaths (19.20%) at decedent's home.

### 3.1 Overall and Sex Stratified Mortality TrendsMortality Trends

**FIGURE 1:**
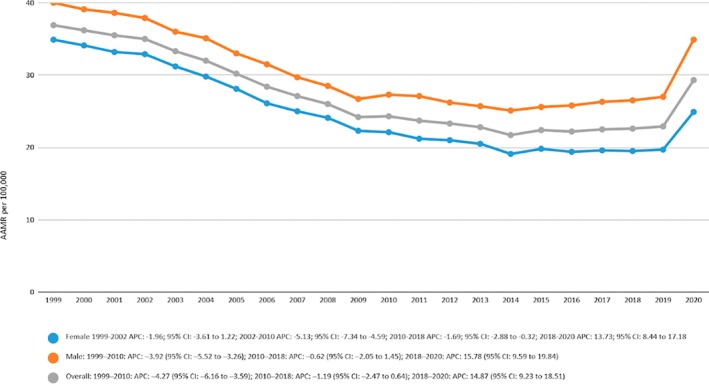
Age‐adjusted mortality rates (AAMRs) per 100,000 individuals stratified by sex/gender in the United States, 1999 to 2020.

The overall mortality trends in CeVD related mortality in patients with Diabetes have been decreasing with an AAMR of 36.9 in 1999 to an AAMR of 29.3 in 2020. Joinpoint regression analysis showed that there was a sharp decrease in annual percentage change (APC) from 1999 to 2010 of −4.2660 (95% CI −6.1582 to −3.585) and then a gradual decrease of −1.1918 (95% CI −2.4668 to 0.6354) and a very sharp increasing trend in mortality from 2018 till 2020 with an APC of 14.8717 (95% CI 9.2308 to 18.5054). Overall, the average annual percentage change (AAPC) was declining with a value of −1.4099 (95% CI −1.7977 to −1.0971). The trend has been shown in the graph in Figure 1 and Table S1.

### 3.2 Age‐Stratified Trends in Mortality

**FIGURE 2:**
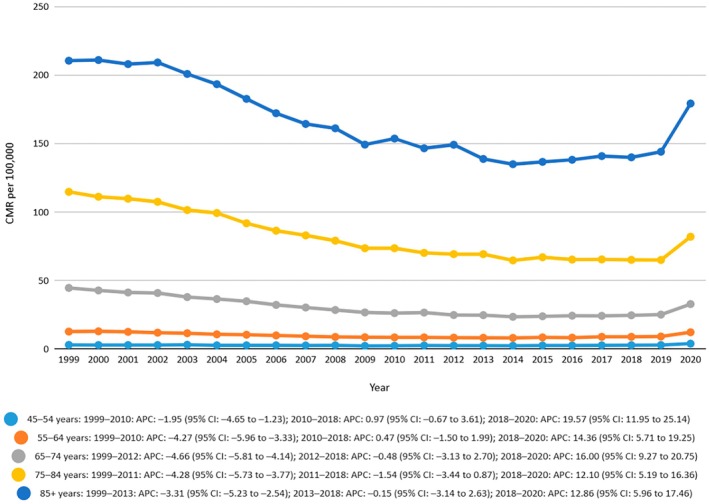
Crude mortality rates (CMRs) per 100,000 individuals stratified by age in the United States, 1999 to 2020.

The crude mortality rate was highest in the age group 75–84 years with a crude mortality rate of 114.8 in 1999 to 82 in 2020. Joinpoint analysis showed a decreasing trend from 1999 to 2011 with an APC value of −4.2836 (95% CI −5.7303 to −3.7684) and also from 2011 to 2018 with an APC of −1.5427 (95% CI −3.4364 to 0.8721) and then a very sharp rise in the trend from 2018 to 2020 with an APC of 12.1010 (95% CI 5.1904 to 16.3562). The AAPC was −1.9136 (95% CI −2.341 to −1.658). The crude mortality rate was lowest among the age group 45–54 years which showed a slight increase from 3 to 3.9 from 1999 to 2020 with an AAPC of 1.0408 (0.5739 to 1.412). The crude rate from 1999 to 2020 for the age group 55–64 years was 9.6 with an AAPC of −0.8230 (−1.4055 to −0.4046). AAPC for the age group 65–74 also showed a declining trend with a crude rate of 29.8 from 1999 to 2020 and an AAPC of −1.6665 (−2.0872 to −1.3789). AAPC for the age group 85+ years was −1.1226 (−1.5521 to −0.8297) and a crude rate of mortality of 162.9 from 1999 to 2020. Age‐related trends in mortality have been shown in Figure 2 and Table S2.

### 3.3 AAMR Stratified by Race

**FIGURE 3:**
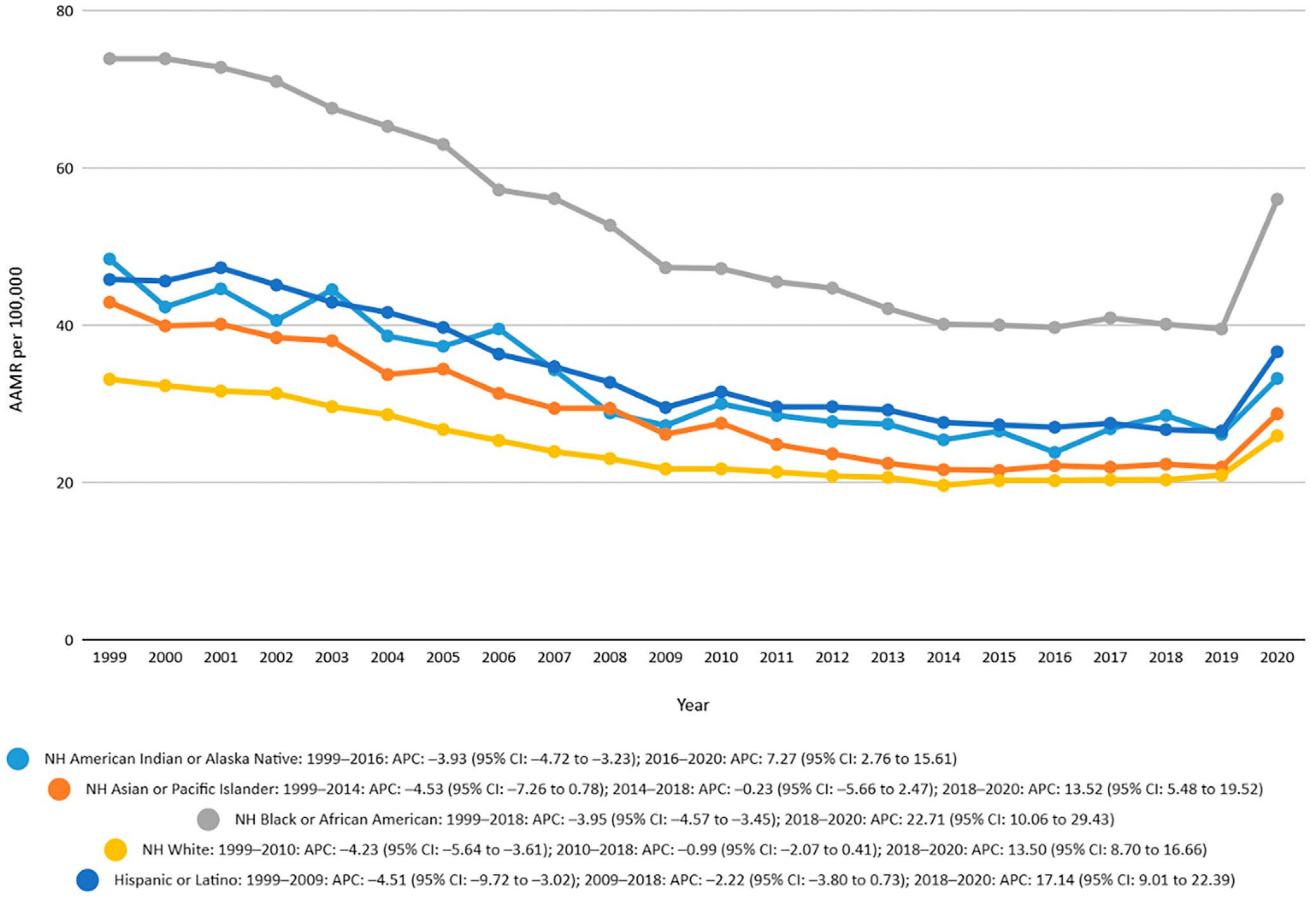
Age‐adjusted mortality rates (AAMRs) per 100,000 individuals stratified by race/ethnicity in the United States, 1999 to 2020.

Mortality trend analysis from 1999 to 2020 showed that the mortality was significantly lower in the White population compared to other races, with an overall AAMR of 33.2 in 1999 and 26 in 2020. Joinpoint analysis showed that there was a decreasing trend from 1999 to 2010 with an APC −4.2321 (−5.6438 to −3.6083) and from 2010 to 2018 an APC of −0.9873 (−2.0655 to 0.40650) and a very sharp rise from 2018 to 2020 with an APC of 13.4969 (8.6958 to 16.6553). The Black population had the highest mortality rate, followed by the Hispanic and then American Indians population, respectively. The mortality rates were lowest among the Asian population, with an AAMR of 27 from 1999 to 2020. The American Indian or Alaskan Native population showed a slow decline in the rate from 1999 to 2016, with an APC of −3.9264 (−4.7239 to −3.2302) and an increase from 2016 to 2020 with an APC of 7.2726 (2.7575–15.6074). The trend is shown in Figure 3 and Table S3.

### 3.4 AAMR Stratified by Urbanisation

**FIGURE 4:**
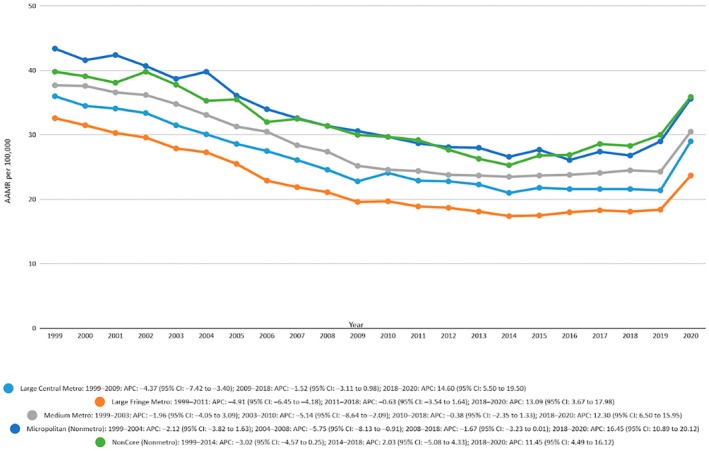
Age‐adjusted mortality rates (AAMRs) per 100,000 individuals stratified by urbanisation in the United States, 1999 to 2020.

Trend for mortality showed that from 1999 to 2020 the AAMR was low in the metropolitan areas which was 14.15 (95% CI 14.1–14.2) compared to non‐metropolitan areas which was 17.84 (95% CI 14.72–14.96). The Joinpoint trend analysis of the metropolitan areas showed that the mortality rate declined consistently among the time intervals 1999–2002, 2002–2009, and 2009–2018 which showed APC values of −0.413, −5.7978, −1.3491 respectively but then it had a sharp increase in the trend from 2018 to 2020 with an APC value of 8.3991 (95% CI 0.84–16.5707). Analysis for non‐metropolitan areas revealed that the mortality trend was decreasing in the interval between 1999 and 2013 with an APC value −3.6658 and similarly between 2103 and 2018 with an APC value −0.0708 but there was a sharp increase in mortality from 2018 to 2020 with an APC value of 8.5499 (95% CI 0.7568–16.9459). The overall AAPC was positively high among the metropolitan areas with AAPC 2.1308 (95% CI 3.0079–1.2458) and the AAPC for the non‐metropolitan areas was 1.7105 (95% CI 2.5754 to −0.838). The results are demonstrated in Figure 4 and Table S4.

### 3.5 Geographical Trends in AAMR

**FIGURE 5:**
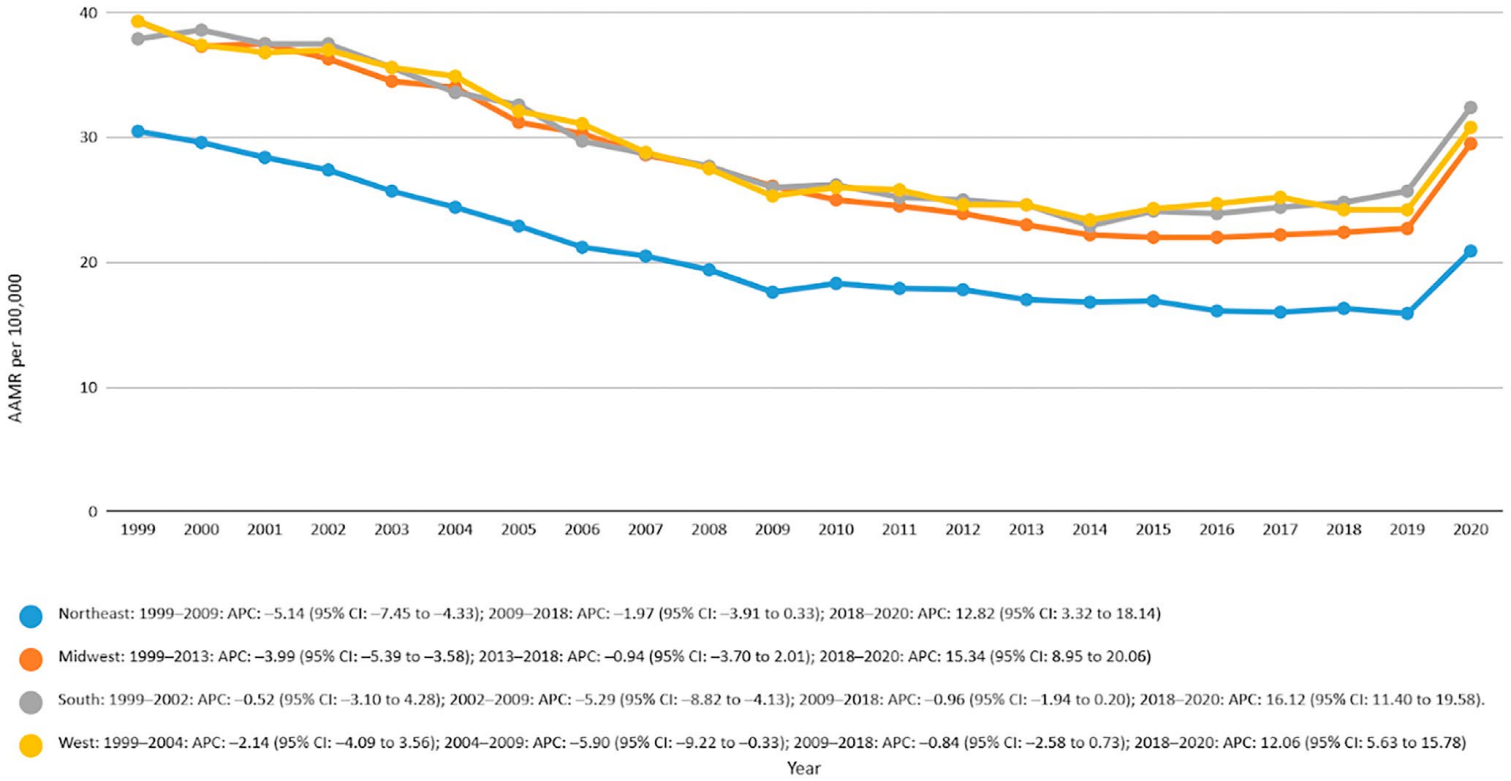
Age‐adjusted mortality rates (AAMRs) per 100,000 individuals stratified by census region in the United States, 1999 to 2020.

AAMR was highest among the region South from 1999 to 2020 with a value of 28.7, followed by an AAMR of 27.8 in the Midwest region, then 26.9 in the West, and was lowest in the Northeast region with an AAMR of 20.5. The overall trend showed a decline in mortality rates with an AAPC of −2.1919 in the Northeast region 95% CI (−2.7691 to −1.8578), the AAPC for the Midwest region was −1.5713 (95% CI −1.9762 to −1.3367), the AAPC for the South region was −0.8747 (95% CI −1.1995 to −0.5209) and the AAPC for the West region was −1.2320 95% CI (−1.6511 to −0.783). The states in the 90th percentile of mortality above the threshold AAMR ≥ 36.25 were Mississippi (AAMR = 44.1), West Virginia (AAMR = 37.9), and Oklahoma (AAMR = 38.3). The states that fall in the 10th percentile of AAMR below the threshold AAMR ≤ 17.83 include Nevada (AAMR = 13.1), Arizona (AAMR = 15.4), Massachusetts (AAMR = 15.6), Florida (AAMR = 16.4), Connecticut (AAMR = 17.3), and New York (AAMR = 17.6). The results are depicted by Figure 5 and Tables S5 and S6.

## 4 Discussion

This study analyzes the trends in CeVD‐related deaths among diabetic patients in the United States from 1999 to 2020. There is an overall decline in the mortality pattern from 1999 to 2018. The mortality rate was highest among 75‐ to 85‐year‐old individuals, the Black population, non‐metropolitan areas, and the South region. However, following 2018, there was an increase in the mortality trend across all demographic and geographic divisions.

Diabetes raises the risk of cerebrovascular death due to complex interactions like persistent hyperglycaemia, which leads to endothelial dysfunction [15]. Chronic hyperglycaemia damages the vascular endothelium and speeds up atherosclerosis in cerebral arteries by promoting oxidative stress [16]. Moreover, insulin resistance promotes dyslipidaemia, causing higher LDL, lower HDL, which exacerbates plaque instability and hence increases the risk of cerebrovascular accidents [17]. Diabetes also causes structural and functional changes in small cerebral vessels at the microvascular level. Prolonged hyperglycaemia causes pericyte loss and, hence, harms the blood–brain barrier, which results in microaneurysms, increasing the risk of both lacunar and microvascular infarcts [18]. Together, all these pathophysiological mechanisms explain the increase in cerebrovascular‐related deaths in patients with diabetes.

According to our study, the AAMR for CeVD‐related mortality decreased from 36.9 (1999) to 29.3 (2020), with a notable decrease from 1999 to 2010 (APC: −4.27%) and an ongoing fall until 2018, followed by a sharp rise in 2018–2020. These findings are consistent with mortality trends due to CeVD, a study conducted in the United States where the mortality rate decreased until 2018 by better stroke management and diabetes care [19]. The decline in the mortality rate was found outside the United States as well; a study conducted in Taiwan, China, also reported a decrease in the death rate due to cerebrovascular events in patients with diabetes [20]. Nevertheless, the increase after 2018 might be due to the COVID‐19 pandemic's disruptions, delayed care, and an increase in the risk of direct vascular damage from SARS‐CoV‐2 infection [21, 22].

The drop prior to 2018 highlights the efficacy of evidence‐based treatments for the management of stroke and diabetes. The rise in deaths after 2018 draws attention to underlying deficiencies made apparent by the pandemic and deteriorating trends in population health. To address these problems and restore care continuity, immediate action is required.

The 75–84 age group had the highest crude mortality rate (114.8 in 1999 to 82 in 2020), which is in line with the idea that comorbidities associated with ageing increase the risk of CeVD in diabetes. These findings align with the Gerardo et al. study, which states that vascular problems are more likely to occur in older diabetic patients. Compared to elders without diabetes, their chance of having a stroke is three times higher. Furthermore, cardiovascular and cerebrovascular events are linked to up to 75%–80% of mortality in diabetic individuals [23]. Younger age groups (45–54 years) had the lowest crude mortality rate (3.9) but revealed a little rise in AAPC (+1.0408). Analysis shows that rising rates of risk factors such as obesity, high blood pressure, high cholesterol, and type 2 diabetes are associated with an increase in the prevalence of stroke among younger persons, hence raising AAPC value a bit [24]. The lowest crude mortality in younger individuals compared to the elderly is due to the fact that long‐term vascular complications of DM take years to develop, hence decreasing crude mortality in young people with diabetes [25]. These patterns highlight the need for age‐appropriate interventions, such as better comorbidity management for older persons and increased primary prevention for younger populations.

Analysis revealed that the rate of cerebrovascular incidents in diabetics declined across all racial and ethnic groups from 1999 to 2020. Asian people had the lowest AAMR, 27, whereas Black populations had the highest CeVD mortality rates, followed by Hispanic, White (AAMR of 33.2), and American Indian/Alaska Native groups from 1999 to 2020. These variations are widely reported. As an example, Saad et al. and other studies documented the same pattern. Blacks or African Americans had the greatest AAMR attributable to cerebrovascular conditions (AAMR 1050; 95% CI), followed by whites, American Indians or Alaska Natives, Hispanics or Latinos, and Asians or Pacific Islanders (AAMR 669.3; 95% CI), as reported by Saad et al. [9]. These disparities are due to different genetic and socioeconomic factors, such as Asian Americans being less prone to experience thrombotic events than Black people, who are substantially more likely to experience them [26]. Also, Black people are less likely to obtain advanced therapies such as mechanical thrombectomy and reperfusion therapy compared to other populations, which may be partially explained by socioeconomic factors [19]. This conversation is important because it shows how acute public health emergencies and systemic disparities interact to shape mortality trends. The results highlight the need for focused interventions and corroborate previous studies on racial differences in diabetes and stroke outcomes. This section urges culturally suitable strategies to close these gaps and places the findings in a larger public health conversation.

Non‐metropolitan areas had higher AAMR (17.84) compared to metropolitan regions [25, 26], likely due to limited healthcare facilities access in rural areas [9]. These findings align with another study that assesses traumatic brain injury fatality rates across different demographics of the United States, that patients with traumatic brain injury (TBI) who live in rural areas with fewer resources are much more likely to die from TBI than those who live in urban areas with more resources [27]. This uneven distribution of healthcare facilities and resources throughout different non‐metropolitan regions of the United States is the leading cause for high AAMR in non‐metropolitan areas [9]. Another retrospective cohort study, employing data from the national inpatient database, explains the disparity between rural and urban areas by pointing out that patients in rural areas with acute stroke are less likely to receive sophisticated therapies such as intravenous thrombolysis and endovascular treatment; hence, they have a higher overall in‐hospital death rate than their urban counterparts [9]. Reducing the mortality rate from CeVD in non‐metropolitan areas requires a multimodal strategy that addresses healthcare access and prevention. Acute care delivery can be enhanced by connecting rural clinicians with stroke specialists through the expansion of telemedicine services, and emergency treatment shortages could be filled by mobile stroke units outfitted with CT scanners. In rural locations, building more stroke‐ready hospitals would guarantee prompt access to endovascular therapy and thrombolysis.

In terms of geography, the Northeast reported the lowest AAMR (20.5), while the South had the highest AAMR (28.7) followed by the Midwest and West. Among the states, California has the highest state‐level mortality rate with an AAMR of 32.8 between 1999 and 2020. Meanwhile, Alaska has the lowest AAMR (23.8). Our results, however, depart from those of another study that found that the Midwest has the highest rate of cerebrovascular‐related mortality, followed by the South and West. Meanwhile, the Northeast had the same lowest AAMR [24]. Merely another study, Geographical Disparity and Traumatic Brain Injury in America, shows results that are consistent with our findings [27]. This enduring regional disparity can be a result of variations in healthcare accessibility, health behaviours, and the regional prevalence of chronic diseases. To lessen this inequality, regional health policy changes that are specific to these vulnerable communities are required.

### 4.1 Limitations

There are various limitations to this study that need to be taken into account. Firstly, we are unable to investigate the precise reasons for elevated mortality in certain populations since the CDC WONDER database only offers mortality data and excludes clinical and molecular factors like lab findings or particular therapeutic measures and genetic traits. Secondly, the small sample sizes for some racial groups may have had an impact on statistical accuracy. Thirdly, risk factors at the individual level, such as socioeconomic status, smoking, and body mass index, are not taken into consideration by the ecological study design. Lastly, the COVID‐19 pandemic may have confused the late mortality increase, particularly in 2020.

## 5 Conclusion

This study provides an in‐depth analysis of the mortality trend from CeVD‐related deaths among diabetics in the United States from 1999 to 2020. Our findings show a notable decrease in AAMRs throughout the first two decades of the 21st century, which is primarily due to advancements in diabetes treatment, acute stroke care, and preventative health measures. However, a reversal of this trend after 2018 across all age and geographic divisions raises deep concern alluding to new issues such as delayed availability of healthcare, disruptions brought on by the pandemic, and perhaps increased comorbidities. Black people, senior citizens, people living in non‐metropolitan areas, and people residing in the Southern United States all had persistently higher mortality rates, highlighting ongoing healthcare disparities. Conversely, the Northeast's people and Asian populations reported the lowest AAMRs. These results highlight how closely demographic, socioeconomic, and geographic factors interact to affect overall mortality outcomes. To lessen the burden of cerebrovascular mortality in diabetic populations, it is crucial to improve access to advanced stroke care, improve the healthcare system, and prioritise preventative measures.

## Author Contributions

**Allahdad Khan:** conceptualization, writing – original draft, writing – review and editing, project administration, investigation. **Waseef Ullah:** writing – original draft. **Moeen Ikram:** visualization, investigation, data curation, formal analysis. **Rameez Qasim:** writing – original draft. **Umama Alam:** writing – original draft. **Maheen Sheraz:** writing – original draft, visualization, software. **Ayesha Khan:** writing – original draft. **Kainat Kanwal:** validation, resources, writing – review and editing. **Peter Collins:** project administration, supervision, methodology, writing – review and editing. **Raheel Ahmed:** supervision, writing – review and editing.

## Conflicts of Interest

The authors declare no conflicts of interest.

## Supporting information


**Data S1:** edm270091‐sup‐0001‐Tables.docx.

## Data Availability

The data that supports the findings of this study are available in Supporting Information of this article.
