# Supplementary material for: Mortality Trends and Disparities in Cerebrovascular Disease Among Diabetic Population in the United States From 1999 to 2020: A CDC WONDER Analysis
Source: Endocrinol Diabetes Metab. 2025 Aug 22;8(5):e70091. doi: 10.1002/edm2.70091 (PMC12371552; doi:10.1002/edm2.70091)
Supplement: Supplementary file 1 — Data S1: edm270091‐sup‐0001‐Tables.docx. [file EDM2-8-e70091-s001.docx]

**Supplementary File:**

**Supplementary Table 1:** Annual overall and sex-stratified age adjusted mortality rates per 100,000 individuals due to diabetes mellitus and cerebrovascular disease in adults aged 45 and above in the United States, 1999 to 2020.

| **Table:** Annual age adjusted mortality rates per 100,000 individuals in adults aged 45+ in the United States, 1999 to 2020 | | | | | |
| --- | --- | --- | --- | --- | --- |
|  |  |  | **Age Adjusted Mortality Rate per 100,000 (95% CI)** | | |
| **Year** | **Deaths** | **Population** | **Overall** | **Females** | **Males** |
| 1999 | 35138 | 95153686 | 36.9 (37.3–36.6) | 34.9 (35.4–34.4) | 40.0 (40.6–39.3) |
| 2000 | 34796 | 96944389 | 36.2 (36.5–35.8) | 34.1 (34.6–33.7) | 39.1 (39.7–38.4) |
| 2001 | 34671 | 99781854 | 35.5 (35.8–35.1) | 33.2 (33.7–32.7) | 38.6 (39.2–37.9) |
| 2002 | 34733 | 102217733 | 35.0 (35.4–34.6) | 32.9 (33.4–32.5) | 37.9 (38.6–37.3) |
| 2003 | 33552 | 104692428 | 33.3 (33.6–32.9) | 31.2 (31.6–30.7) | 36.0 (36.6–35.4) |
| 2004 | 32773 | 107138553 | 32.0 (32.4–31.7) | 29.8 (30.3–29.4) | 35.1 (35.7–34.5) |
| 2005 | 31525 | 109787199 | 30.2 (30.5–29.9) | 28.1 (28.5–27.7) | 33.0 (33.6–32.5) |
| 2006 | 30218 | 112380379 | 28.4 (28.7–28.1) | 26.1 (26.5–25.7) | 31.5 (32.0–31.0) |
| 2007 | 29349 | 114894084 | 27.1 (27.4–26.8) | 25.0 (25.4–24.6) | 29.7 (30.2–29.2) |
| 2008 | 28752 | 117395131 | 26.0 (26.3–25.7) | 24.1 (24.5–23.7) | 28.5 (29.0–28.0) |
| 2009 | 27373 | 119895863 | 24.2 (24.5–23.9) | 22.3 (22.7–22.0) | 26.7 (27.2–26.2) |
| 2010 | 27951 | 121757429 | 24.3 (24.6–24.0) | 22.1 (22.5–21.7) | 27.3 (27.7–26.8) |
| 2011 | 28072 | 124174484 | 23.7 (24.0–23.5) | 21.2 (21.6–20.9) | 27.1 (27.6–26.7) |
| 2012 | 28194 | 126000296 | 23.3 (23.6–23.0) | 21.0 (21.3–20.6) | 26.2 (26.7–25.8) |
| 2013 | 28260 | 127788037 | 22.8 (23.1–22.5) | 20.5 (20.8–20.2) | 25.7 (26.1–25.2) |
| 2014 | 27660 | 129779643 | 21.7 (22.0–21.5) | 19.1 (19.4–18.8) | 25.1 (25.6–24.7) |
| 2015 | 29079 | 131826832 | 22.4 (22.6–22.1) | 19.8 (20.1–19.4) | 25.6 (26.1–25.2) |
| 2016 | 29631 | 133494018 | 22.2 (22.5–22.0) | 19.4 (19.7–19.1) | 25.8 (26.2–25.4) |
| 2017 | 30799 | 135229289 | 22.5 (22.8–22.3) | 19.6 (19.9–19.3) | 26.3 (26.7–25.9) |
| 2018 | 31602 | 136335528 | 22.6 (22.8–22.3) | 19.5 (19.8–19.2) | 26.5 (26.9–26.1) |
| 2019 | 32849 | 137381702 | 22.9 (23.2–22.7) | 19.7 (20.0–19.4) | 27.0 (27.4–26.5) |
| 2020 | 42869 | 138429175 | 29.3 (29.6–29.0) | 24.9 (25.2–24.5) | 34.9 (35.3–34.4 |

**Supplementary Table 2:** Annual Crude mortality rates (CMRs) per 100,000 individuals stratified by age groups due to diabetes mellitus and cerebrovascular disease in adults aged 45 and above in the United States, 1999 to 2020

| **Table:** Annual Crude mortality rates (CMRs) per 100,000 individuals in adults aged 45+ in the United States, 1999 to 2020 | | | | | |
| --- | --- | --- | --- | --- | --- |
|  | **Crude mortality rates (CMRs) per 100,000 (95% CI) stratified by age-groups** | | | | |
| **Year** | **45-54 years** | **55-64 years** | **65-74 years** | **75-84 years** | **85+ years** |
| 1999 | 3.0 (3.2-2.8) | 12.7 (13.2-12.3) | 44.6 (45.6-43.7) | 114.8 (116.7-112.9) | 210.6 (215.1-206.2) |
| 2000 | 2.9 (3.1-2.7) | 12.9 (13.4-12.5) | 42.8 (43.7-41.8) | 111.2 (113.1-109.3) | 211.1 (215.5-206.8) |
| 2001 | 2.9 (3.1-2.8) | 12.5 (12.9-12.0) | 41.3 (42.2-40.3) | 109.8 (111.6-107.9) | 208.1 (212.4-203.8) |
| 2002 | 2.9 (3.1-2.8) | 11.9 (12.3-11.5) | 40.9 (41.8-40.0) | 107.5 (109.3-105.7) | 209.3 (213.5-205.0) |
| 2003 | 3.1 (3.2-2.9) | 11.5 (11.9-11.1) | 37.9 (38.8-37.0) | 101.5 (103.2-99.8) | 200.9 (205.0-196.7) |
| 2004 | 2.7 (2.9-2.6) | 10.7 (11.0-10.3) | 36.5 (37.4-35.7) | 99.3 (101.0-97.6) | 193.4 (197.4-189.4) |
| 2005 | 2.7 (2.9-2.6) | 10.4 (10.8-10.1) | 34.9 (35.7-34.1) | 91.8 (93.4-90.1) | 182.7 (186.6-178.9) |
| 2006 | 2.7 (2.8-2.5) | 9.9 (10.3-9.6) | 32.2 (33.0-31.4) | 86.4 (88.0-84.8) | 172.2 (175.9-168.5) |
| 2007 | 2.6 (2.8-2.5) | 9.3 (9.6-9.0) | 30.3 (31.1-29.6) | 83.0 (84.5-81.4) | 164.4 (167.9-160.8) |
| 2008 | 2.7 (2.8-2.5) | 8.8 (9.1-8.5) | 28.5 (29.2-27.7) | 79.1 (80.6-77.6) | 161.2 (164.7-157.8) |
| 2009 | 2.3 (2.5-2.2) | 8.6 (8.9-8.3) | 26.7 (27.4-26.0) | 73.6 (75.1-72.2) | 149.3 (152.6-146.0) |
| 2010 | 2.4 (2.5-2.3) | 8.5 (8.8-8.2) | 26.2 (26.9-25.6) | 73.6 (75.1-72.1) | 153.7 (156.9-150.4) |
| 2011 | 2.6 (2.8-2.5) | 8.5 (8.8-8.2) | 26.6 (27.3-25.9) | 70.2 (71.7-68.8) | 146.6 (149.7-143.5) |
| 2012 | 2.5 (2.6-2.3) | 8.3 (8.5-8.0) | 24.8 (25.4-24.2) | 69.3 (70.7-67.9) | 149.2 (152.3-146.1) |
| 2013 | 2.5 (2.6-2.3) | 8.2 (8.5-7.9) | 24.7 (25.3-24.1) | 69.3 (70.7-67.9) | 138.8 (141.8-135.8) |
| 2014 | 2.4 (2.6-2.3) | 8.1 (8.4-7.8) | 23.5 (24.1-22.9) | 64.7 (66.0-63.3) | 135.0 (137.9-132.1) |
| 2015 | 2.6 (2.7-2.4) | 8.5 (8.8-8.2) | 23.9 (24.5-23.3) | 67.0 (68.3-65.6) | 136.7 (139.6-133.8) |
| 2016 | 2.6 (2.8-2.5) | 8.3 (8.6-8.0) | 24.3 (24.9-23.8) | 65.3 (66.6-63.9) | 138.2 (141.1-135.3) |
| 2017 | 2.7 (2.8-2.5) | 8.9 (9.2-8.6) | 24.2 (24.8-23.7) | 65.4 (66.7-64.1) | 140.9 (143.8-138.0) |
| 2018 | 2.8 (2.9-2.6) | 8.9 (9.2-8.6) | 24.6 (25.1-24.0) | 65.1 (66.4-63.9) | 140.0 (142.9-137.1) |
| 2019 | 2.9 (3.0-2.7) | 9.1 (9.4-8.8) | 25.1 (25.7-24.6) | 65.0 (66.3-63.8) | 144.1 (147.0-141.2) |
| 2020 | 3.9 (4.1-3.7) | 12.2 (12.5-11.9) | 32.8 (33.5-32.2) | 82.0 (83.4-80.6) | 179.3 (182.5-176.1) |

**Supplementary Table 3:** Annual race-stratified age adjusted mortality rates per 100,000 individuals due to diabetes mellitus and cerebrovascular disease in adults aged 45 and above in the United States, 1999 to 2020

| **Table:** Annual age adjusted mortality rates per 100,000 individuals in adults aged 45+ in the United States, 1999 to 2020 | | | | | |
| --- | --- | --- | --- | --- | --- |
|  | **Race-Stratified Age Adjusted Mortality Rate per 100,000 (95% CI)** | | | | |
| **Year** | **American Indian/Alaska Native** | **Asian/Pacific Islander** | **Black/African American** | **White** | **Hispanic/Latino** |
| 1999 | 48.5 (55.6-41.3) | 43.0 (46.0-40.1) | 74.0 (75.9-72.1) | 33.2 (33.6-32.8) | 45.9 (48.0-43.8) |
| 2000 | 42.4 (48.6-36.1) | 40.0 (42.7-37.2) | 74.0 (75.9-72.1) | 32.4 (32.8-32.0) | 45.7 (47.8-43.7) |
| 2001 | 44.7 (51.1-38.4) | 40.2 (42.9-37.6) | 72.9 (74.7-71.0) | 31.7 (32.0-31.3) | 47.4 (49.4-45.4) |
| 2002 | 40.7 (46.5-34.8) | 38.5 (41.1-36.0) | 71.1 (72.9-69.3) | 31.4 (31.8-31.0) | 45.2 (47.1-43.3) |
| 2003 | 44.6 (50.8-38.5) | 38.1 (40.5-35.7) | 67.7 (69.4-65.9) | 29.7 (30.0-29.3) | 43.0 (44.8-41.2) |
| 2004 | 38.7 (44.3-33.2) | 33.8 (36.0-31.6) | 65.4 (67.2-63.7) | 28.7 (29.0-28.3) | 41.7 (43.4-39.9) |
| 2005 | 37.4 (42.8-32.0) | 34.5 (36.6-32.3) | 63.1 (64.8-61.4) | 26.8 (27.1-26.5) | 39.8 (41.4-38.2) |
| 2006 | 39.6 (45.1-34.0) | 31.4 (33.4-29.4) | 57.3 (58.9-55.7) | 25.4 (25.7-25.0) | 36.4 (37.9-34.8) |
| 2007 | 34.4 (39.4-29.4) | 29.5 (31.3-27.6) | 56.2 (57.7-54.6) | 24.0 (24.3-23.7) | 34.8 (36.2-33.3) |
| 2008 | 28.9 (33.3-24.4) | 29.5 (31.3-27.7) | 52.8 (54.3-51.4) | 23.1 (23.5-22.8) | 32.8 (34.2-31.4) |
| 2009 | 27.3 (31.4-23.2) | 26.2 (27.8-24.5) | 47.4 (48.8-46.0) | 21.8 (22.1-21.5) | 29.6 (30.8-28.3) |
| 2010 | 30.1 (34.5-25.8) | 27.6 (29.3-25.9) | 47.3 (48.6-45.9) | 21.8 (22.1-21.5) | 31.6 (32.8-30.3) |
| 2011 | 28.6 (32.6-24.6) | 24.9 (26.4-23.4) | 45.6 (47.0-44.3) | 21.4 (21.7-21.1) | 29.7 (30.9-28.5) |
| 2012 | 27.8 (31.7-23.9) | 23.7 (25.1-22.2) | 44.8 (46.1-43.5) | 20.9 (21.2-20.6) | 29.7 (30.9-28.6) |
| 2013 | 27.5 (31.2-23.8) | 22.5 (23.9-21.2) | 42.2 (43.5-41.0) | 20.7 (20.9-20.4) | 29.3 (30.4-28.1) |
| 2014 | 25.5 (29.0-22.0) | 21.7 (23.0-20.4) | 40.2 (41.4-39.0) | 19.7 (19.9-19.4) | 27.7 (28.7-26.6) |
| 2015 | 26.6 (30.1-23.2) | 21.6 (22.8-20.4) | 40.1 (41.2-38.9) | 20.3 (20.6-20.1) | 27.4 (28.5-26.4) |
| 2016 | 23.9 (27.1-20.7) | 22.2 (23.4-21.0) | 39.8 (40.9-38.6) | 20.3 (20.5-20.0) | 27.1 (28.1-26.1) |
| 2017 | 26.9 (30.2-23.6) | 22.0 (23.2-20.9) | 41.0 (42.1-39.9) | 20.4 (20.6-20.1) | 27.6 (28.6-26.6) |
| 2018 | 28.6 (31.9-25.4) | 22.4 (23.6-21.3) | 40.2 (41.3-39.1) | 20.4 (20.7-20.2) | 26.8 (27.7-25.8) |
| 2019 | 26.2 (29.2-23.2) | 22.0 (23.1-20.9) | 39.6 (40.7-38.6) | 21.0 (21.2-20.7) | 26.6 (27.5-25.7) |
| 2020 | 33.3 (36.5-30.0) | 28.8 (30.0-27.5) | 56.1 (57.3-54.8) | 26.0 (26.3-25.8) | 36.7 (37.7-35.6) |

**Supplementary Table 4:** Annual age adjusted mortality rates per 100,000 individuals stratified by 2013 urbanisation due to diabetes mellitus and cerebrovascular disease in adults aged 45 and above in the United States, 1999 to 2020

| **Table:** Annual age adjusted mortality rates per 100,000 individuals in adults aged 45+ in the United States, 1999 to 2020 | | | | | | |
| --- | --- | --- | --- | --- | --- | --- |
|  | **Urbanisation-Stratified Age Adjusted Mortality Rate per 100,000 (95% CI)** | | | | | |
| **Year** | **Large Central Metro** | **Large Fringe Metro** | **Medium Metro** | **Small Metro** | **Micropolitan (Nonmetro)** | **NonCore (Nonmetro)** |
| 1999 | 36.0 (36.7-35.3) | 32.6 (33.4-31.8) | 37.7 (38.5-36.8) | 38.9 (40.1-37.6) | 43.4 (44.7-42.1) | 39.8 (41.2-38.5) |
| 2000 | 34.5 (35.2-33.8) | 31.5 (32.3-30.8) | 37.6 (38.4-36.7) | 40.0 (41.3-38.8) | 41.6 (42.9-40.4) | 39.1 (40.5-37.8) |
| 2001 | 34.1 (34.8-33.4) | 30.3 (31.0-29.5) | 36.6 (37.4-35.8) | 39.1 (40.4-37.9) | 42.4 (43.7-41.1) | 38.1 (39.4-36.7) |
| 2002 | 33.4 (34.1-32.7) | 29.6 (30.3-28.9) | 36.2 (37.0-35.4) | 39.2 (40.4-37.9) | 40.7 (42.0-39.5) | 39.8 (41.2-38.5) |
| 2003 | 31.5 (32.2-30.9) | 27.9 (28.6-27.2) | 34.8 (35.6-34.0) | 37.6 (38.8-36.4) | 38.7 (39.9-37.6) | 37.8 (39.1-36.4) |
| 2004 | 30.1 (30.8-29.5) | 27.3 (27.9-26.6) | 33.1 (33.8-32.3) | 35.4 (36.6-34.3) | 39.8 (41.0-38.6) | 35.3 (36.6-34.0) |
| 2005 | 28.6 (29.2-27.9) | 25.5 (26.1-24.9) | 31.3 (32.0-30.6) | 33.2 (34.3-32.1) | 36.1 (37.2-34.9) | 35.5 (36.7-34.2) |
| 2006 | 27.5 (28.1-26.9) | 22.9 (23.5-22.3) | 30.5 (31.2-29.7) | 30.9 (32.0-29.9) | 34.0 (35.1-32.9) | 32.0 (33.2-30.8) |
| 2007 | 26.1 (26.7-25.5) | 21.9 (22.5-21.3) | 28.4 (29.1-27.7) | 29.5 (30.6-28.5) | 32.6 (33.7-31.5) | 32.5 (33.7-31.3) |
| 2008 | 24.6 (25.2-24.0) | 21.1 (21.7-20.5) | 27.4 (28.1-26.8) | 28.5 (29.5-27.5) | 31.4 (32.4-30.4) | 31.4 (32.5-30.2) |
| 2009 | 22.8 (23.3-22.3) | 19.6 (20.1-19.0) | 25.2 (25.8-24.5) | 26.3 (27.2-25.3) | 30.6 (31.6-29.6) | 30.0 (31.2-28.9) |
| 2010 | 24.1 (24.6-23.5) | 19.7 (20.2-19.2) | 24.6 (25.2-24.0) | 26.2 (27.1-25.2) | 29.7 (30.7-28.7) | 29.7 (30.8-28.5) |
| 2011 | 22.9 (23.5-22.4) | 18.9 (19.4-18.4) | 24.4 (25.0-23.8) | 27.3 (28.2-26.3) | 28.7 (29.6-27.7) | 29.2 (30.3-28.1) |
| 2012 | 22.8 (23.3-22.3) | 18.7 (19.2-18.2) | 23.8 (24.4-23.2) | 26.3 (27.2-25.4) | 28.1 (29.0-27.1) | 27.7 (28.7-26.6) |
| 2013 | 22.3 (22.8-21.7) | 18.1 (18.6-17.7) | 23.7 (24.3-23.1) | 26.0 (26.9-25.1) | 28.0 (29.0-27.1) | 26.3 (27.4-25.3) |
| 2014 | 21.0 (21.5-20.5) | 17.4 (17.9-17.0) | 23.5 (24.0-22.9) | 23.1 (24.0-22.3) | 26.6 (27.5-25.7) | 25.3 (26.4-24.3) |
| 2015 | 21.8 (22.2-21.3) | 17.5 (18.0-17.1) | 23.7 (24.3-23.1) | 24.5 (25.3-23.6) | 27.7 (28.6-26.7) | 26.8 (27.9-25.8) |
| 2016 | 21.6 (22.1-21.1) | 18.0 (18.5-17.6) | 23.8 (24.4-23.3) | 23.8 (24.7-23.0) | 26.1 (27.0-25.2) | 26.9 (27.9-25.8) |
| 2017 | 21.6 (22.1-21.1) | 18.3 (18.8-17.8) | 24.1 (24.7-23.6) | 23.1 (23.9-22.3) | 27.4 (28.3-26.5) | 28.6 (29.7-27.6) |
| 2018 | 21.6 (22.1-21.1) | 18.1 (18.6-17.7) | 24.5 (25.0-23.9) | 24.2 (25.0-23.4) | 26.8 (27.7-25.9) | 28.3 (29.4-27.3) |
| 2019 | 21.4 (21.9-21.0) | 18.4 (18.9-18.0) | 24.3 (24.9-23.8) | 24.4 (25.3-23.6) | 29.0 (29.9-28.1) | 30.0 (31.0-28.9) |
| 2020 | 29.0 (29.5-28.5) | 23.7 (24.2-23.2) | 30.5 (31.2-29.9) | 31.1 (32.0-30.1) | 35.6 (36.6-34.6) | 35.9 (37.1-34.8) |

**Supplementary Table 5:** Annual age adjusted mortality rates per 100,000 individuals stratified by census region due to diabetes mellitus and cerebrovascular disease in adults aged 45 and above in the United States, 1999 to 2020

| **Table:** Annual age adjusted mortality rates per 100,000 individuals in adults aged 45+ in the United States, 1999 to 2020 | | | | |
| --- | --- | --- | --- | --- |
|  | **Census Region-Stratified Age Adjusted Mortality Rate per 100,000 (95% CI)** | | | |
| **Year** | **Northeast AAMR (95% CI)** | **Midwest AAMR (95% CI)** | **South AAMR (95% CI)** | **West AAMR (95% CI)** |
| 1999 | 30.5 (29.8–31.3) | 39.3 (38.5–40.1) | 37.9 (37.2–38.6) | 39.3 (38.4–40.2) |
| 2000 | 29.6 (28.8–30.3) | 37.3 (36.5–38.1) | 38.6 (37.9–39.3) | 37.4 (36.5–38.3) |
| 2001 | 28.4 (27.6–29.1) | 37.5 (36.7–38.3) | 37.5 (36.8–38.1) | 36.8 (36.0–37.7) |
| 2002 | 27.4 (26.7–28.1) | 36.3 (35.5–37.0) | 37.5 (36.9–38.2) | 37.0 (36.1–37.8) |
| 2003 | 26.3 (25.6–27.0) | 35.1 (34.3–35.9) | 36.3 (35.6–36.9) | 36.2 (35.4–37.0) |
| 2004 | 25.7 (25.0–26.4) | 34.5 (33.7–35.2) | 35.6 (34.9–36.2) | 35.6 (34.7–36.4) |
| 2005 | 24.8 (24.2–25.5) | 33.2 (32.5–34.0) | 34.8 (34.1–35.5) | 34.6 (33.8–35.4) |
| 2006 | 24.1 (23.5–24.8) | 32.1 (31.3–32.8) | 34.0 (33.3–34.7) | 33.6 (32.8–34.4) |
| 2007 | 23.4 (22.7–24.0) | 31.0 (30.3–31.7) | 33.2 (32.6–33.9) | 32.6 (31.8–33.4) |
| 2008 | 22.8 (22.2–23.4) | 30.2 (29.5–30.9) | 32.5 (31.9–33.1) | 31.7 (30.9–32.5) |
| 2009 | 22.3 (21.7–22.9) | 29.5 (28.8–30.1) | 31.9 (31.3–32.5) | 30.8 (30.0–31.6) |
| 2010 | 21.8 (21.2–22.4) | 28.7 (28.1–29.4) | 31.2 (30.6–31.8) | 29.9 (29.1–30.7) |
| 2011 | 21.3 (20.7–21.9) | 28.0 (27.4–28.6) | 30.6 (30.0–31.2) | 29.0 (28.2–29.8) |
| 2012 | 20.8 (20.2–21.4) | 27.3 (26.7–27.9) | 30.0 (29.4–30.6) | 28.1 (27.3–28.9) |
| 2013 | 20.3 (19.7–20.9) | 26.6 (26.0–27.2) | 29.4 (28.8–30.0) | 27.2 (26.4–28.0) |
| 2014 | 19.8 (19.2–20.4) | 25.9 (25.3–26.5) | 28.8 (28.2–29.4) | 26.3 (25.5–27.1) |
| 2015 | 19.3 (18.8–19.9) | 25.2 (24.7–25.8) | 28.2 (27.6–28.8) | 25.4 (24.6–26.2) |
| 2016 | 18.9 (18.4–19.4) | 24.5 (24.0–25.1) | 27.7 (27.1–28.3) | 24.6 (23.8–25.4) |
| 2017 | 18.4 (17.9–18.9) | 23.9 (23.3–24.4) | 27.1 (26.6–27.7) | 23.8 (23.0–24.6) |
| 2018 | 17.9 (17.4–18.4) | 23.2 (22.7–23.7) | 26.6 (26.0–27.1) | 23.0 (22.2–23.8) |
| 2019 | 17.4 (17.0–17.9) | 22.6 (22.1–23.1) | 26.0 (25.5–26.6) | 22.2 (21.4–23.0) |
| 2020 | 16.9 (16.5–17.4) | 21.9 (21.4–22.4) | 25.5 (25.0–26.0) | 21.4 (20.6–22.2) |

**Supplementary Table 6:** Overall age-adjusted mortality rates per 100,000 individuals stratified by states due to diabetes mellitus and cerebrovascular disease in adults aged 45 and above in the United States, 1999 to 2020

| **Table:** Annual age adjusted mortality rates per 100,000 individuals in adults aged 45+ in the United States, 1999 to 2020 | | |
| --- | --- | --- |
| **State** | **Age Adjusted Mortality Rate per 100,000 (95% CI)** | **% of Total Deaths** |
| Alabama | 29.6 (30.2-29.1) | 1.70% |
| Alaska | 25.3 (27.1-23.4) | 0.10% |
| Arizona | 15.4 (15.7-15.0) | 1.20% |
| Arkansas | 28.7 (29.4-28.1) | 1.10% |
| California | 32.8 (33.0-32.6) | 13.10% |
| Colorado | 22.3 (22.8-21.8) | 1.10% |
| Connecticut | 17.3 (17.8-16.9) | 0.80% |
| Delaware | 22.6 (23.6-21.5) | 0.30% |
| District of Columbia | 34.3 (36.0-32.6) | 0.20% |
| Florida | 16.4 (16.6-16.2) | 4.90% |
| Georgia | 23.9 (24.3-23.5) | 2.20% |
| Hawaii | 29.0 (29.9-28.1) | 0.50% |
| Idaho | 25.8 (26.8-24.9) | 0.40% |
| Illinois | 23.2 (23.4-22.9) | 3.50% |
| Indiana | 30.3 (30.8-29.9) | 2.30% |
| Iowa | 27.0 (27.6-26.4) | 1.20% |
| Kansas | 22.6 (23.2-22.0) | 0.80% |
| Kentucky | 32.1 (32.7-31.5) | 1.70% |
| Louisiana | 28.3 (28.8-27.7) | 1.50% |
| Maine | 23.0 (23.8-22.2) | 0.40% |
| Maryland | 31.0 (31.5-30.5) | 2.00% |
| Massachusetts | 15.6 (15.9-15.3) | 1.40% |
| Michigan | 25.0 (25.4-24.7) | 3.10% |
| Minnesota | 30.6 (31.1-30.1) | 2.00% |
| Mississippi | 44.1 (45.0-43.3) | 1.50% |
| Missouri | 25.3 (25.8-24.9) | 1.90% |
| Montana | 24.3 (25.3-23.3) | 0.30% |
| Nebraska | 30.1 (31.0-29.3) | 0.70% |
| Nevada | 13.1 (13.6-12.6) | 0.40% |
| New Hampshire | 22.4 (23.3-21.5) | 0.40% |
| New Jersey | 20.8 (21.2-20.5) | 2.30% |
| New Mexico | 22.0 (22.7-21.3) | 0.50% |
| New York | 17.6 (17.8-17.4) | 4.40% |
| North Carolina | 34.8 (35.2-34.3) | 3.80% |
| North Dakota | 29.9 (31.2-28.6) | 0.30% |
| Ohio | 35.6 (35.9-35.2) | 5.30% |
| Oklahoma | 38.3 (38.9-37.6) | 1.70% |
| Oregon | 35.4 (36.0-34.7) | 1.70% |
| Pennsylvania | 25.7 (26.0-25.5) | 4.80% |
| Rhode Island | 24.3 (25.3-23.4) | 0.40% |
| South Carolina | 35.6 (36.2-35.0) | 2.00% |
| South Dakota | 29.0 (30.2-27.8) | 0.30% |
| Tennessee | 35.5 (36.0-35.0) | 2.70% |
| Texas | 34.7 (35.0-34.4) | 8.30% |
| Utah | 20.9 (21.7-20.2) | 0.50% |
| Vermont | 35.7 (37.2-34.1) | 0.30% |
| Virginia | 25.1 (25.5-24.7) | 2.20% |
| Washington | 31.9 (32.4-31.4) | 2.40% |
| West Virginia | 37.9 (38.8-37.0) | 1.00% |
| Wisconsin | 25.6 (26.0-25.2) | 1.90% |
| Wyoming | 22.2 (23.6-20.8) | 0.10% |
